# Less Common Causes of Acute Abdomen

**DOI:** 10.5334/jbr-btr.1045

**Published:** 2016-02-16

**Authors:** Lieven Van Hoe, Filip Claus, Piet Vanhoenacker

**Affiliations:** 1 OLVZ Aalst, BE

In this presentation, less common causes of acute abdomen are reviewed. Emphasis is on three topics. First, the anatomy of the superior mesenteric vein and mesentery is reviewed, together with the anatomical basis and clinical/radiological presentation of midgut volvulus. Emphasis is on diagnostic clues (whirl sign, abnormal position of cecum, small bowel, and third part of duodenum) and pitfalls (positional variation of small bowel loops with pseudo-twisting of mesenteric vessels). Second, atypical presentations of acute appendicitis and appendagitis, as well as their mimics, are discussed. Finally, an overview is given of uncommon but important causes of free intraperitoneal fluid in patients with acute abdominal pain.

## Competing Interests

The authors declare that they have no competing interests.

